# Editorial Notes and Comments

**Published:** 1906-01

**Authors:** 


### EDITORIAL NOTES AND COMMENTS.

We are much pleased to be able to announce that *Dental Hints,* which has been edited by Dr. B. H. Teague for the past seven years or more, has been united with the JOURNAL. Dr. Teague becomes one of the editors of the JOURNAL and takes charge of a new department to be known as The Dental Hints Department.

Dr. Teague has proven to be a very popular editor and we are proud to have him connected with the JOURNAL.

---

All unexpired subscriptions to *Dental Hints* will receive the JOURNAL for the remainder of the term for which they have paid—and we trust they will then renew for another year.

---

Dr. W. R. Pearson, of Reidsville, Ga., writes us that he wishes to sell his practice as he is going into another line of business. Any one wishing to purchase a good practice should write him.

---

The Lawrence Amalgam was placed on the market in 1846 and is now celebrating its sixtieth birthday.

The fact that it has been able to hold its business under the fierce competition of the many alloys on the market today, speaks well for the quality of this amalgam.

---

We are in receipt of a letter from a subscriber asking the formula of Watt's Metal, an alloy that was used to support artificial teeth before the use of rubber. Will somebody kindly inform our friend.

---

We wish to secure a copy of the February, 1905, issue of the *Dental Brief*. A year's subscription to the JOURNAL will be given in payment.

---

It's the worker who succeeds in life—not the chap who is worked.

---

Any fool can spend money; some fools can make it; but the fool who can make and keep it cheats folly and becomes wise.

---

A man who stands square with the world stands on the foundation of his fortune.

---

A wise man who made a little improvement each day found at the end of the year a revolution in his business.

---

Men who use perfume ought to be chloroformed at any old age.

---

One man takes his work as a stone around his neck and sinks to apathy. Another takes it as a stepping stone and mounts to success.

